# A deep‐learning‐based pipeline for automatic fusion of CT coronary angiogram and stress perfusion CMR

**DOI:** 10.1002/mp.70420

**Published:** 2026-04-10

**Authors:** Wenting Jiang, Ming‐Yen Ng, Tsun‐Hei Sin, Peng Cao

**Affiliations:** ^1^ Department of Diagnostic Radiology The University of Hong Kong Hong Kong China

**Keywords:** cardiac MRI, CT coronary angiogram, deep learning, image fusion

## Abstract

**Background:**

Accurate evaluation of coronary artery constriction and myocardial ischemia is essential for diagnosing and managing coronary artery disease (CAD). Combining CT coronary angiography (CTCA) and stress cardiovascular magnetic resonance (CMR) imaging allows examination of both coronary artery narrowing and myocardial perfusion.

**Purpose:**

To develop a deep learning pipeline that integrates CTCA and CMR images, which could help improve accuracy in identifying affected vessels and their associated myocardial territories.

**Methods:**

The proposed pipeline included two deep learning models: one for automatic reorientation of 3D CTCA and another for left ventricle (LV) wall registration between CTCA and CMR images. A 3D spatial co‐registration model, the reorientation spatial transformer network (Reorientation STN), predicted reorientation parameters for input CTCA volumes using ResNet18 and STN. A 2D nonrigid spatial deformation network (Nonrigid SDN) was trained for LV wall registration. Cross‐modal supervision was employed during training. Evaluation criteria included aspect ratio (AR), Dice similarity coefficient (DSC), and long‐axis deviation angles. The process involved quantifying LV wall perfusion on registered CMR images and extracting coronary arteries from reoriented CTCA images to fuse these results. The pipeline was trained and validated on 447 pairs of CTCA and CMR images from 75 patients and tested on 18 subjects.

**Results:**

The pipeline achieved an AR of 0.94 ± 0.03, long‐axis deviation angles of 1.19 ± 0.83 (axial) and 1.54 ± 0.79 (coronal), a DSC of 0.66 ± 0.04 for LV wall reorientation, and a DSC of 0.92 ± 0.03 for LV wall registration between CTCA and CMR.

**Conclusions:**

This automated framework successfully fuses cardiac CTCA and CMR imaging, demonstrating its potential effectiveness.

## 1 INTRODUCTION

A precise evaluation of coronary artery narrowing and myocardial ischemia is crucial for the identification and management of coronary artery disease (CAD). By combining CT coronary angiography (CTCA) and stress cardiovascular magnetic resonance (CMR) imaging, clinicians can assess both the anatomical narrowing of coronary arteries and myocardial perfusion, providing a comprehensive view of coronary circulation and the downstream consequences of obstructive CAD.^1^ Traditionally, medical practitioners manually correlated affected coronary artery segments with their respective myocardial territories based on the American Heart Association 17‐segment model.^2^ The advent of 3D fusion methodologies has enabled the integration of coronary structure and myocardial perfusion data, significantly improving the accuracy of vessel‐to‐territory assignment.^3^, ^4^ While the fusion of CTCA and CMR images has been studied using traditional medical image processing algorithms.^3^, ^4^, ^5^ These techniques often necessitate manual segmentation, registration, and fusion interventions. Recent progress in artificial intelligence (AI) has facilitated the development of fully automated frameworks for CTCA and CMR integration, reducing reliance on manual input.^6^, ^7^ In particular, deep learning models have shown promise in automating cardiac tissue segmentation and perfusion quantification, expediting clinical workflows, and improving risk prediction.^8^, ^9^, ^10^, ^11^ Despite these advancements and the reported clinical benefits of deep learning in cardiovascular imaging, there is still a lack of research on the automatic fusion of CTCA and CMR images using deep learning methodologies.

Traditionally, CTCA images were obtained as transaxial slices perpendicular to the patient's longitudinal axis but not aligned with the left ventricle's (LV) long axis.^12^ Conversely, CMR images were procured as short‐axis slices aligned with LV's long axis, a view vital for precise LV quantification and myocardial perfusion analysis.^13^ Ensuring a thorough diagnostic interpretation of cardiac CTCA and CMR images necessitated reorienting the initial CTCA images to match the short‐axis view. This reorientation enabled accurate LV quantification and detailed myocardial perfusion assessment,^14^ and it facilitated the efficient fusion and integration of CTCA and CMR data.^15^ Deep learning techniques have resulted in significant advancements in reorienting cardiac single‐photon emission computed tomography (SPECT) images, achieving outstanding performance.^16^, ^17^, ^18^, ^19^ By establishing a consistent anatomical reference through CTCA reorientation, reliable LV quantification, robust wall perfusion analysis, and seamless integration of CTCA and CMR data for comprehensive diagnostic interpretation can be achieved.

Building on these developments, we proposed an automated deep‐learning‐based pipeline for integrating 3D CTCA and CMR images. This framework enabled a direct correlation between coronary artery constriction and stress‐induced LV wall hypoperfusion, offering valuable insights for the identification and management of CAD.

## 2 MATERIALS AND METHODS

### 2.1 Data acquisition

This study included 93 patients who underwent both CTCA and stress CMR imaging at local medical imaging centers to train and evaluate the proposed deep neural networks. CTCA examinations were performed using the Philips iCT256 scanner, following locally established standard protocols at a single site. CTCA data acquisition involved administering iodinated contrast based on patient weight. Spiral acquisition captured continuous volumetric data. Bolus tracking ensured precise image acquisition during contrast passage. Other imaging parameters were as follows: voxel size of 0.625 × 0.625 × 0.625 mm, image dimension of 512 × 512, slices of 241–389. CMR examinations were performed using the GE Signa Premier 3T scanner. Data acquisition based on T1‐weighted gradient echo was used for CMR perfusion. Details of the imaging protocol for stress CMR acquisition have been described previously in our earlier study.^20^ CMR imaging was performed on a 3.0T SIGNA Premier scanner. For anatomical imaging, parameters included a field of view (FOV) of 32–38 × 24–28 cm, matrix size of 192 × 148, number of excitations (NEX) of 0.75, flip angle of 15°, repetition time (TR) ranging from 2.6 to 3.1 ms, echo time (TE) ranging from 1.2 to 1.5 ms, and a parallel imaging acceleration factor of 2. For the stress perfusion protocol, imaging was performed with an FOV of 36–40 cm, phase FOV of 0.75–0.80, matrix size of 192 × 148, flip angle of 20°, TR of 3.4 ms, TE of 1.1–1.5 ms, NEX of 0.75, and slice thickness of 8 mm. The contrast agent used was Gadoterate meglumine at a dose of 0.05 mmol/kg, followed by a saline flush of 30 mL at 3 mL/s. Adenosine at 140 µg/kg/min was administered over 3–4 min as the stress agent, with 75 mg of aminophylline used as the reversal agent. Perfusion sequence was initiated after the patient achieved adequate stress. Adequate stress was defined as a heart rate rise >10 bpm, systolic blood pressure rise >10 mmHg, or symptoms compatible with stress response (e.g., chest pain, shortness of breath, etc.). In this study, CMR imaging parameters included three short‐axis slices and 90 time frames. Additional imaging details are provided in our previous study.^20^

### 2.2 Data preprocessing

The patient cohort was subdivided into two subgroups: Group 1, 75 patients, designated for model training and validation, and Group 2, 18 patients, designated for model testing. The dataset encompassed 447 pairs of data collected from 75 patients, including CTCA images captured at five various phases between 45 and 80% of the R‐R interval. Additional CTCA images were generated by randomly rotating the images along the *X*, *Y*, and *Z* axes, ranging from −180° to 180°, with a fixed seed assigned to each sample. This procedure mimics the practical situation in which reorienting CTCA images to the short‐axis view always requires rotations within 180° along each axis on our dataset. Integrating the transformed images with the previously obtained 447 pairs of data increased the total number of datasets to 2235 pairs. Each pair contained an input CTCA image and the corresponding referenced CMR image (last time point), providing valuable multimodal guidance during the model training process. Additionally, one short‐axis view CTCA template was obtained by manually reorienting the CTCA images. This involved aligning the LV two‐chamber (vertical) long axis orthogonally through the apex and mitral valve, as well as the four‐chamber (horizontal) long axis orthogonally through the mitral valve, left atrium, and LV long axis (21). The CMR dataset was curated by selecting the last 10 time points exhibiting the highest contrast from the initial collection of 90 time points. This procedure resulted in the construction of a CMR dataset comprising 750 CMR images. Data augmentation techniques, such as flipping and rotation with randomly assigned parameter values for each epoch during the training process, were employed to further enrich the CTCA dataset. Data preprocessing techniques, including resampling, min–max normalization, and cropping, were applied to the CTCA and CMR datasets. Additionally, motion correction was performed by rigidly registering the last 10 time points of the CMR to the last time point on the CMR dataset, which exhibited the optimal contrast for the LV and LV wall. This study received approval from the IRB under the reference number UW19‐0100.

### 2.3 Deep‐learning‐based pipeline

The proposed framework employed deep learning to process acquired CTCA and CMR images. The steps to generate the fused images included pseudo‐label CTCA generation for CTCA reorientation, pseudo‐label CMR generation for CMR LV wall registration with CTCA, and deployment of deep learning models to fuse CTCA and CMR data.

**FIGURE 1:**
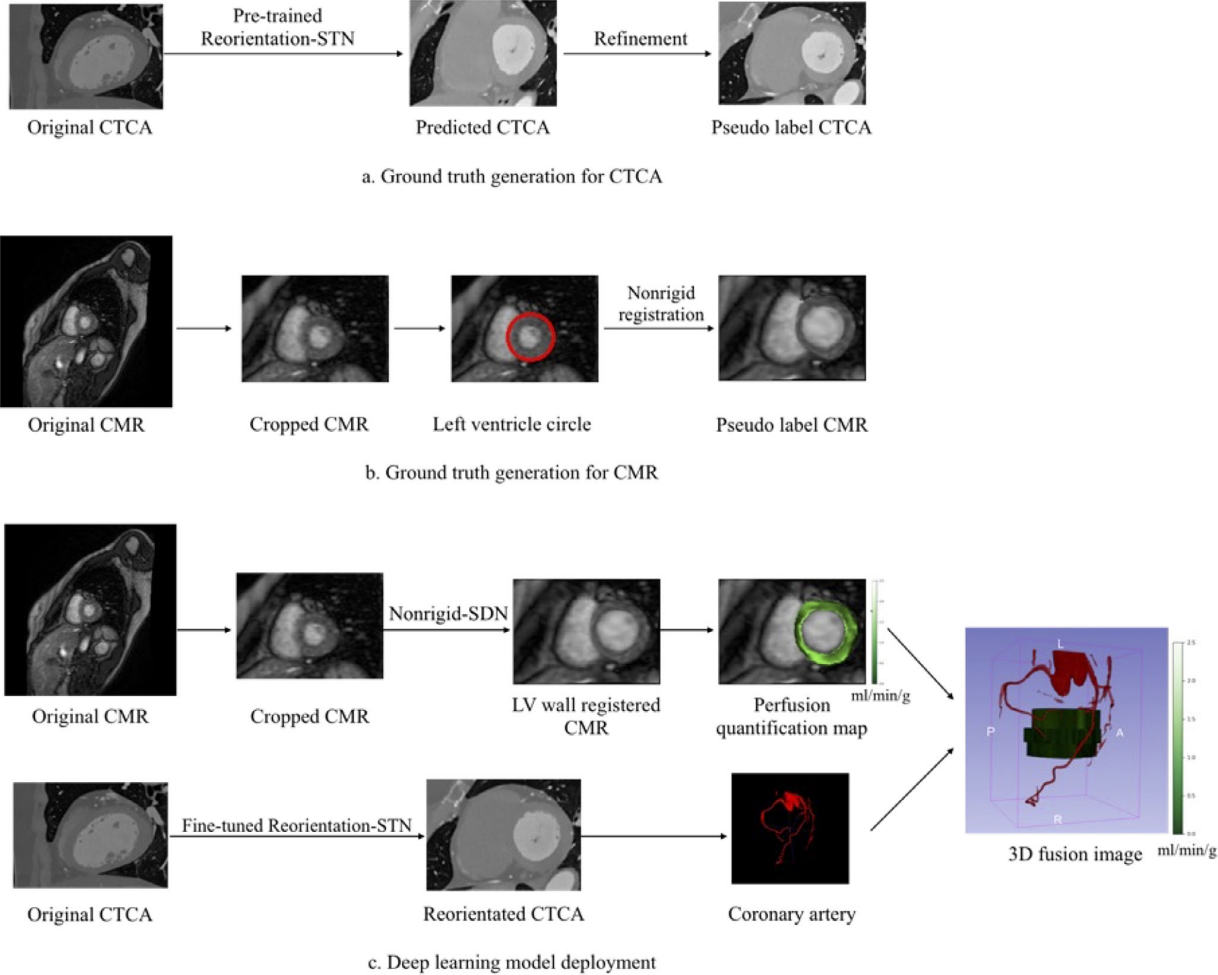
Overview of the automatic fusion pipeline for CTCA and cardiac MRI. Step A: Reorient CTCA images using a pretrained reorientation STN and anatomical prior refinement. Step B: Crop CMR images based on the left ventricle (LV) wall center, followed by nonrigid registration with reoriented CTCA images. Step C: Fine‐tune the reorientation STN with Step A outputs, then train a nonrigid SDN model for registration of cropped CMR and reoriented CTCA. During inference, reorient CTCA using the fine‐tuned model, extract coronary arteries with TotalSegmentator, and use the SDN model to predict LV wall registered CMR images. Perfusion on the registered CMR images is quantified using a physics‐informed neural network. Finally, generate a fused 3D image of the coronary arteries and perfusion map.

**FIGURE 2:**
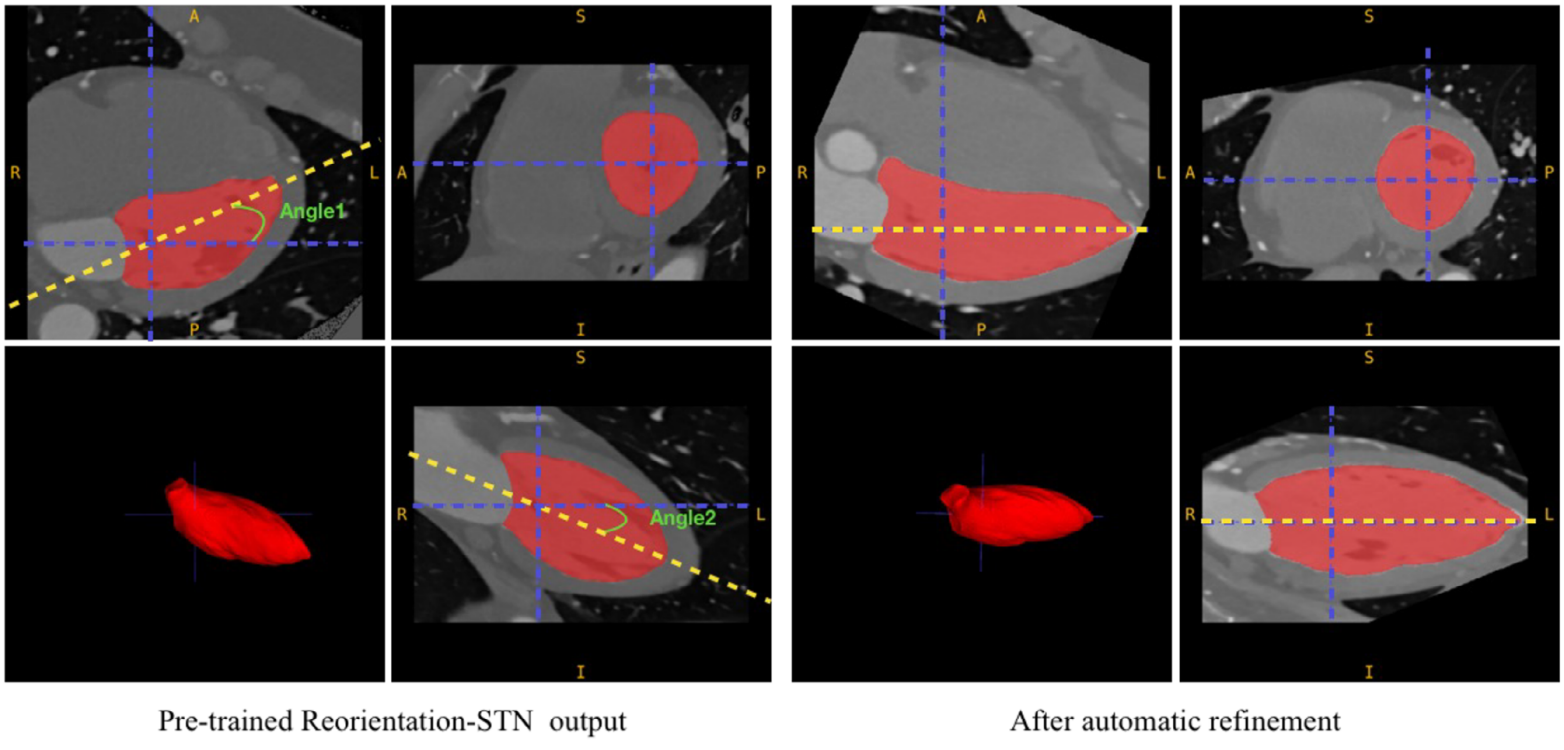
The pseudo‐label generation process for CTCA reorientation, in which the heart's anatomical structure was automatically refined. The LV mask is shown in red, the blue dashed lines delineate the center of LV in the short‐axis view slices, the yellow dashed line indicates the long axis of the LV in axial and coronal planes, and the green curve represents two corresponding long‐axis deviation angles.

The overview of our pipeline for the automatic fusion of CTCA and cardiac MRI is shown in Figure 1. The pipeline comprises several key steps to achieve a precise and comprehensive fusion of the two modalities. In Step A, the original CTCA images were reoriented into a short‐axis view using a pretrained reorientation model called reorientation STN under self‐supervised learning. Subsequently, the predicted CTCA results underwent a refinement process involving an automatic alignment procedure that aligned LV's long axis along the right–left (RL) direction in the axial and coronal planes. The alignment process was facilitated by utilizing the LV mask generated by TotalSegmentator,^21^ which provides automatic segmentations of major cardiac anatomical structures, including the LV, right ventricle (RV), myocardium, and coronary arteries, on our CTCA images using the NVIDIA GeForce RTX 3090 GPU. The TotalSegmentator model was employed off‐the‐shelf, with its pretrained weights and without further retraining. This LV mask was applied to the short‐axis view slice in the sagittal plane to achieve the desired alignment (Figure 2). To determine the long axis, the short‐axis view slice indicated by the crosshair in Figure 2 in the sagittal plane was selected initially. Subsequently, two lines were delineated: one connecting the center of the LV to the LV apex (the rightmost point from the LV mask) in the axial plane and the other in the coronal plane. The long axis of the LV was then aligned along the RL direction in both the axial and coronal planes (Figure 2). Following this alignment procedure, the pseudo‐label CTCA was generated (Figure 1a).

Step B involved the processing of the original CMR images. Initially, the original CMR images were cropped to a fixed FOV of 105 × 75, utilizing the center of the LV circle detected by the Hough transform, focusing on the heart region. In our study, stress perfusion CMR images were acquired using ECG gating, typically at different cardiac phases than those used for cardiac CTCA. This discrepancy in timing can lead to observed variations in the measurement of LV wall thickness between the two imaging modalities. To address this discrepancy, a nonrigid registration approach was employed to ensure consistent LV wall thickness between the two modalities. This registration technique was applied to the LV wall masks of the CMR and CTCA images, which were generated automatically using trained deep learning models. Segment CMR,^22^ a specialized software solution for quantitative cardiac MR analysis, was employed for CMR images, while TotalSegmentator,^21^ which provides automatic segmentations of major cardiac anatomical structures, was utilized for CTCA images. Finally, to ensure consistent LV wall thickness preservation across the CMR and CTCA modalities, a nonrigid registration methodology was implemented on the LV wall binary mask of CMR and CTCA. This deformable registration approach incorporated six pyramid levels and a grid regularization parameter of 0.6. Then, the pseudo‐label CMR was generated (Figure 1b).

In Step C (model deployment), the reorientation STN was fine‐tuned under the supervision of the pseudo‐label CTCA obtained in Step A. Additionally, a spatial deformation network (SDN) was trained to achieve nonrigid registration between the cropped CMR images and the reoriented CTCA images. Furthermore, a nonrigid SDN model was trained under the supervision of pseudo‐label CMR obtained in Step B, as well as the cropped CMR and reoriented CTCA images as inputs. During the inference process, the original CTCA images were reoriented into a short‐axis view using the fine‐tuned reorientation STN model. The coronary arteries were then extracted from the reoriented CTCA images using the TotalSegmentator.^21^ Simultaneously, the nonrigid SDN model was employed to predict the registered CMR images to maintain consistency with CTCA. LV wall perfusion on registered CMR images was quantified using a physics‐informed neural network.^23^ Finally, the three‐dimensional (3D) rendering fusion of the coronary arteries from CTCA and perfusion quantification maps from CMR was performed using the 3D Slicer software.^24^ This process facilitated the generation of a 3D visualization of the fused images, enabling comprehensive examination in a 3D format.

### 2.4 Deep neural network design

The spatial transformer network (STN) is a deep learning module that can be integrated into neural network architectures to enable two‐dimensional spatial transformations of the network's feature map.^25^ Comprising three submodules, namely, a localization network, a grid generator, and a sampler, STN predicts transformation matrices, generates sampling grids to determine the transformed output points, and applies the transformation to the input, respectively. The STN can learn optimal transformation parameters without ground‐truth transformation information.^25^ Notably, the transformation matrix in the STN includes a translation matrix (T) and a rotation matrix (R), which are typically applied to the feature map rather than the original input images.

**FIGURE 3:**
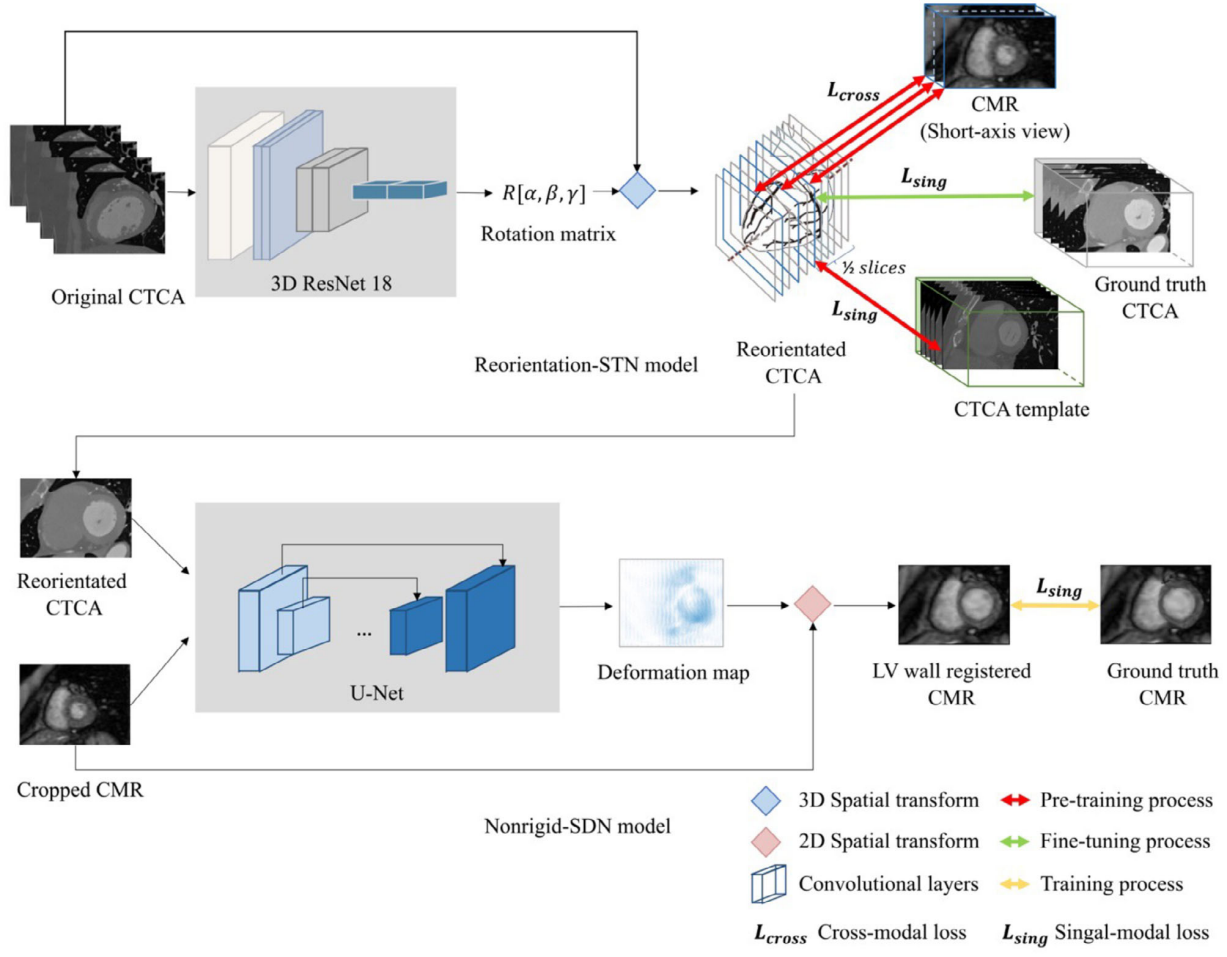
Architecture of the deep learning models. Upper part: The reorientation STN uses ResNet18 to predict reorientation parameters from CTCA, which are then applied via a 3D spatial transformer to generate reoriented CTCA images. Cross‐modal supervision is applied during training. Lower part: The nonrigid SDN model takes reoriented CTCA and cropped CMR as inputs, using a U‐Net to predict a deformation map for registering CMR to CTCA via a 2D spatial transformer.

In this study, we proposed a self‐supervised network named reorientation STN for CTCA reorientation (Figure 3). Utilizing the ResNet18 module, reorientation parameters were predicted based on the input CTCA volume, forming a rotation matrix denoted as *R* [*α*, *β*, *γ*], as described in Equation (1). To ensure compatibility with the subsequent 3D grid generator and sampler in the STN, the rotation matrix *R* was converted from predicted Euler angles to World Coordinate System angles. Notably, the translation matrix was excluded from Equation (1) as our focus was solely on CTCA reorientation without incorporating translational transformations. The resulting rotation matrix *R* served as the input to the spatial transformer function, which transformed the input CTCA images into reoriented output images.

(1) $$\begin{array}{cc} R(\alpha ,\beta ,\gamma ) & =R_{y}\left(\alpha \right)R_{x}\left(\beta \right)R_{y}\left(\gamma \right)=\left[\begin{array}{ccc} \cos\alpha & 0 & \sin\alpha \\ 0 & 1 & 0\\ -\sin\alpha & 0 & \cos\alpha \end{array}\right]\\ & \left[\begin{array}{ccc} 1 & 0 & 0\\ 0 & \cos\beta & -\sin\beta \\ 0 & \sin\beta & \cos\beta \end{array}\right]\left[\begin{array}{ccc} \cos\gamma & -\sin\gamma & 0\\ \sin\gamma & \cos\gamma & 0\\ 0 & 0 & 1 \end{array}\right] \end{array}$$

The 3D reorientation STN network was designed to learn optimal reorientation parameters for CTCA reorientation, even in the absence of clear information on transformation. Additionally, we introduced a novel method that incorporates short‐axis CMR images as cross‐modal supervision for the middle slice of the reoriented CTCA. This cross‐modal supervision provided guidance in learning CTCA reorientation (Figure 3).

During the reorientation STN training process, we employed a loss function comprising two types of image losses. The first type was called single‐modal loss (symbolized as $L_{sing}$), which was calculated between the reoriented CTCA and the CTCA template. The second type was called cross‐modal loss (symbolized as $L_{cross}$), which was calculated between the reoriented CTCA and the corresponding CMR image from the same patient. To compute cross‐modal loss, the short‐axis view slice of the reoriented CTCA volume in the sagittal plane corresponds to the first slice of the corresponding CMR in the short‐axis view (Figure 3). The remaining two slices were selected and separated by a specific length based on CMR slice spacing to align with subsequent CMR slices. This mapping strategy ensured proper alignment between the CTCA and CMR images, ensuring the reoriented CTCA image aligned with the apical‐basal direction and making the calculation of cross‐modal loss more effective.

These losses were measured using the mean squared error (MSE) metric, with different weights assigned to each loss term to reflect their relative importance. The MSE losses for the images were defined as follows:

(2) $$L_{sing}=\frac{1}{n}\sum_{i=1}^{n}{\left(P_{i}-T_{i}\right)}^{2}\;$$

(3) $$L_{cross}=\frac{1}{n}\sum_{i=1}^{n}{\left(P_{i}-R_{i}\right)}^{2}\;$$

where $P_{i}$, $T_{i}$, and $R_{i}$ represent the reoriented CTCA image using the predicted reorientation parameters for the *i*th sample, the fixed manually rotated CTCA template, and the corresponding CMR image for the *i*th sample, respectively. The variable *n* represented the total number of samples in the dataset. To calculate the overall loss, we summed the total loss ($L_{total}$) using the following formulation:

(4) $$L_{total}=\mu_{1}*L_{sing}+\mu_{2}*L_{cross}$$

Here, $\mu_{1}$ and $\mu_{2}$ are empirical parameters that were set to 0.6 and 0.4, respectively, to achieve a proper balance between the two types of losses. The weight $\mu_{1}$ corresponded to the single‐modal loss $L_{sing}$, and $\mu_{2}$ represented the weight assigned to the cross‐modal loss $L_{cross}$. These loss weights *μ*_1 _= 0.6 (single‐modality alignment) and *μ*_2 _= 0.4 (cross‐modality alignment) were chosen empirically. Preliminary tuning with other combinations (e.g., 0.5/0.5, 0.7/0.3) showed that 0.6/0.4 provided the most stable convergence and best validation performance.

The total loss ($L_{total}$) combined the single‐and cross‐modal loss using their respective weights, providing an overall measure of the difference between the reoriented CTCA images and the CTCA template and the corresponding CMR images. This optimization approach ensured accurate CTCA reorientation while leveraging both single‐ and cross‐modal information within the reorientation STN. During the fine‐tuning process of the reorientation STN, the single‐modal loss calculated between reoriented CTCA and pseudo‐label CTCA generated in Step A of Figure 1 was employed.

The architecture of the 2D nonrigid SDN model for nonrigid registration between cropped CMR and reoriented CTCA is shown in Figure 3. It used concatenated short‐axis view slice in reoriented CTCA and cropped CMR as multichannel inputs, which served as the fixed and moving images, respectively. The U‐Net was then employed to predict the deformation map to register the moving CMR to the fixed CTCA using a 2D spatial transformer function. The single‐modal loss calculated between the LV wall registered CMR and pseudo‐label CMR generated in Step B of Figure 1 was employed during the training process.

### 2.5 Experiments

Our experiments were conducted using the NVIDIA GeForce RTX 3090 GPU, along with PyTorch 1.7.1 and CUDA 11.0 for efficient computation. During the training processes, the average time taken to predict CTCA reorientation for each step was 0.74 ± 0.03 s. In the testing phase, the average time for predicting CTCA reorientation on the testing cohort was 0.3389 ± 0.0004 s/sample. ResNet18 was employed as the backbone architecture, and the Adam optimizer with an initial learning rate of 1e − 4 was applied for optimization.

**FIGURE 4:**
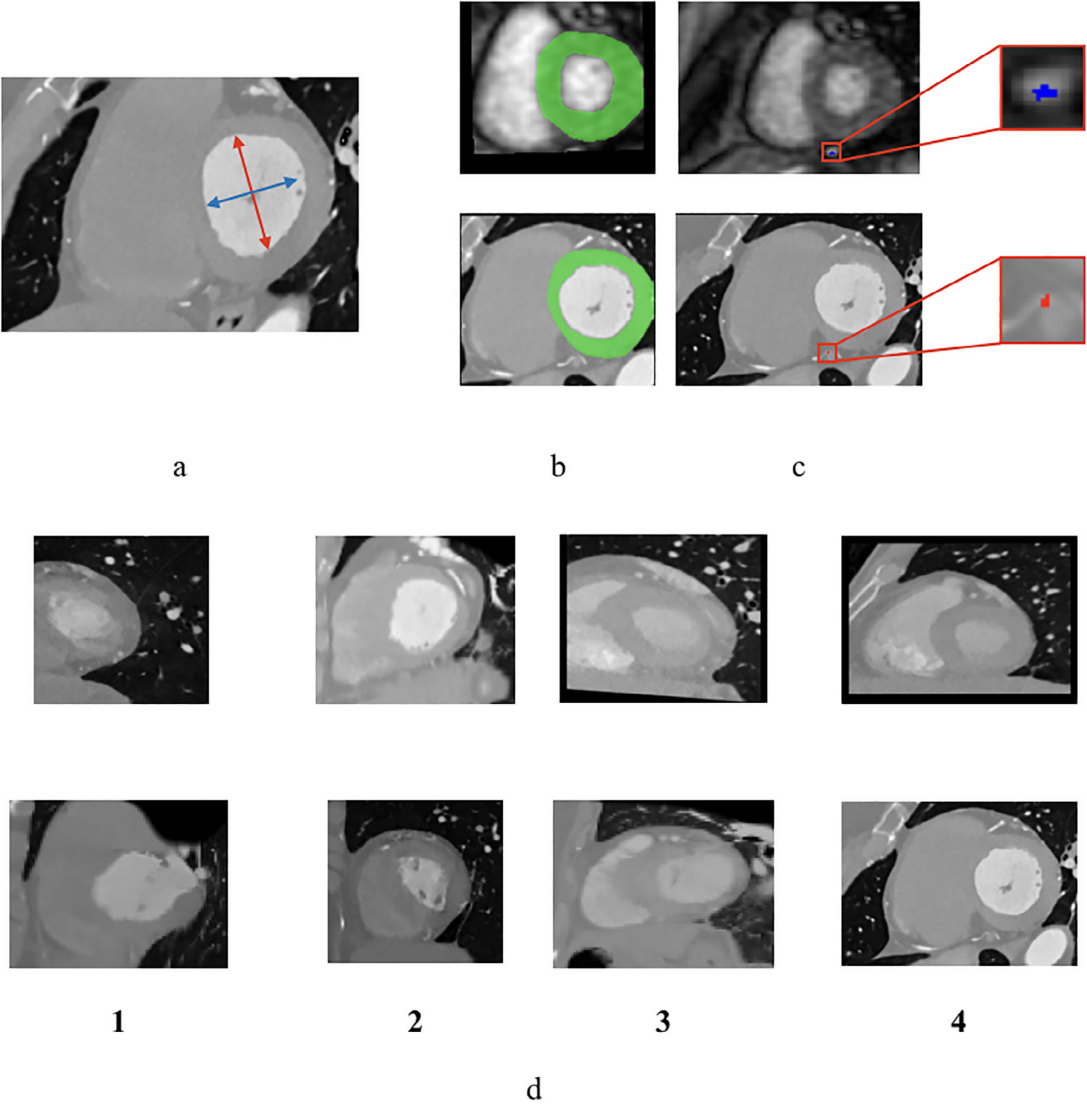
Evaluation criteria for cardiac imaging. (a) Long‐ and short‐axis components of the LV, used to compute the aspect ratio (AR). (b) Myocardial wall masks on CTCA and CMR for Dice similarity coefficient (DSC). (c) Coronary arteries on CMR and CTCA. (d) Four‐point scale for visual assessment of resemblance to the standard short‐axis view (1–4, from absent to close resemblance of key structures to MRI).

To evaluate the performance of the model, we conducted a thorough analysis from three perspectives. First, we calculated the aspect ratio (AR),^26^ which measured the long‐ and short‐axis ratio of the LV in the reoriented CTCA image (Figure 4a). AR indicated the degree of similarity between the reoriented CTCA and the ground‐truth CTCA, specifically in the short‐axis view. An AR closer to 1 signified a higher level of alignment between the reoriented CTCA and the targeted ground‐truth CTCA in a short‐axis view, indicating improved accuracy in reproducing the anatomical structure of the LV. Second, we computed the Dice similarity coefficient (DSC) of the LV wall between the reoriented CTCA image and the corresponding CMR image (Figure 4b). The DSC quantitatively measured the overlap or similarity between the two images, specifically capturing the cross‐modal correspondence of the LV wall. A higher DSC score reflected a more precise alignment of the LV wall between the reoriented CTCA image and the corresponding CMR image, indicating improved consistency and agreement between the two imaging modalities. Third, we quantified the long‐axis deviation angles (LA Dev ANG) between the long axis of the LV in the reoriented CTCA images and the RL direction in both the axial and coronal planes (Figure 2). This quantitative assessment provided an indication of the degree of deviation between the reoriented results and the standard short‐axis view ground truth in the apical‐basal direction. These evaluation metrics were employed to conduct a comprehensive assessment of the model performance, capturing both the accuracy of anatomical alignment in the short‐axis view (measured by AR and LA Dev ANG) and the consistency of LV wall alignment across modalities (measured by DSC). This rigorous evaluation framework provided valuable insights into the model accuracy regarding reorienting CTCA images. The three quantitative metrics (AR, DSC, and LA Dev ANG) were computed on a per‐slice basis and subsequently averaged across all slices of each volume, thereby reflecting both slice‐level alignment and overall volumetric consistency.

For model evaluation, we utilized VoxelMorph,^27^ a widely used learning‐based framework for deformable, pairwise medical image registration. This model was trained on paired original CTCA images and a reoriented CTCA template, enabling the generation of deformation fields for image alignment. The experimental settings were consistent with our proposed reorientation STN. VoxelMorph served as a benchmark to evaluate the effectiveness of our approach in achieving accurate CTCA reorientation and alignment. Furthermore, to validate our model in comparison to the benchmark, we conducted a qualitative assessment using a panel of one senior cardiac radiologist with over 10 years of experience in cardiac imaging, one clinical radiologist with 6 years of experience, and two MRI scientists of 4‐year experience each. One MRI scientist independently performed manual reorientation of the CTCA images. All four experts then independently reviewed the reoriented CTCA and the corresponding CMR images, focusing on the alignment of anatomical structures, particularly the LV and surrounding myocardial tissue. The experts were blinded to the source of the images (model‐generated or original) to ensure unbiased evaluation. They provided qualitative scores based on anatomical fidelity, boundary precision, and clinical usability using a four‐point Likert scale, where 1 = *Poor*, 2 = *Fair*, 3 = *Good*, and 4 = *Excellent*. Figure 4d illustrates a visual example of the four‐point Likert scale (1 = *Poor*, 4 = *Excellent*) criteria, where higher scores denote a greater resemblance to the standard short‐axis view compared to the MRI short‐axis view. Each column corresponds to a specific score: a score of 1 indicates the absence of basic structures (LV, RV, myocardium); a score of 2 indicates the presence of basic structures with significant deviations from the MRI short‐axis view; a score of 3 indicates complete structures with partial resemblance to the corresponding MRI; and a score of 4 indicates complete structures with close resemblance to the corresponding MRI. In cases of disagreement, the average of the experts’ scores was used for analysis. Inte‐expert variability in scoring was assessed using the intraclass correlation coefficient (ICC) across all four reviewers

## 3 RESULTS

The quantitative comparative results of our model for CTCA reorientation and registration to CMR on the testing cohort are presented in Table 1. Our model exhibited higher numerical performance relative to the baseline method VoxelMorph across all evaluated metrics and achieved results that aligned with those obtained through manual reorientation by human experts. The results revealed an AR of 0.94 ± 0.03, LA Dev ANGs of 1.19 ± 0.83 and 1.54 ± 0.79 on reoriented CTCA, a DSC of 0.66 ± 0.04 between reoriented CTCA and cropped CMR, and a DSC of 0.92 ± 0.03 between reoriented CTCA and LV wall registered CMR. These results supported the effectiveness of our model in reorienting CTCA images by considering both anatomical features and cross‐modal correspondence. Our approach showed improved metrics compared to VoxelMorph, with an AR of 0.73 ± 0.13 and DSC of 0.23 ± 0.04, attaining the highest mean values for AR and DSC. Our method achieved the highest AR and DSC values among the benchmark models (VoxelMorph, TransMorph, XMorpher), suggesting precise reorientation and increased overlap of the LV wall between CTCA and CMR images.

**TABLE 1:** Quantitative comparison with state‐of‐the‐art methods on the testing cohort.

Method	AR	DSC	LA Dev ANG1 (°)	LA Dev ANG2 (°)	Time (s/sample)
VoxelMorph^27^	0.73	0.23	30.36	19.11	0.0668
TransMorph^44^	0.68	0.21	21.71	15.95	0.1660
XMorpher^45^	0.42	0.27	38.73	67.92	0.5465
ResNet‐STN (pretrain)	0.79	0.51	24.03	16.31	0.4031
ResNet‐STN (fine‐tune)	**0.94**	**0.66**	**1.19**	**1.54**	0.3389
Manual ground truth	**0.91**	**0.62**	**0**.	**0**.	**518.334**
LV wall registration	‐	**0.92**	‐	‐	‐

*Note*: AR measures the long‐ and short‐axis ratio of the LV. A ratio closer to 1 signifies a higher level of reorientation. Dice similarity coefficient (DSC) measures the overlap of the LV wall on CTCA and CMR. A value closer to 1 indicates better overlap. The final row represents the result of our pipeline.

Abbreviations: AR, aspect ratio; DSC, Dice similarity coefficient; LA Dev ANG, long‐axis deviation angle.

Our model also achieved performance within the range of manual results obtained by human experts (AR = 0.91, DSC = 0.62), with an average inference time of 0.3389 ± 0.0004 seconds per sample. In contrast, human experts required 8.6 ± 2.3 minutes per sample. To further evaluate the robustness of these findings, statistical analyses were performed using 95% confidence intervals and paired Wilcoxon signed‐rank tests (Table S1). The proposed method demonstrated statistically significant improvements over all baseline approaches (*p* < 0.01), indicating that the observed differences are supported by statistical evidence. To further evaluate robustness, a dedicated subgroup analysis was performed on *N* = 7 cases from the testing cohort with abnormal left ventricular morphology (hypertrophy or dilation). This analysis indicated consistent performance levels, with the subgroup achieving a mean DSC of 0.59 and HD = 7.7 mm, which was comparable to that of the overall cohort.

**FIGURE 5:**
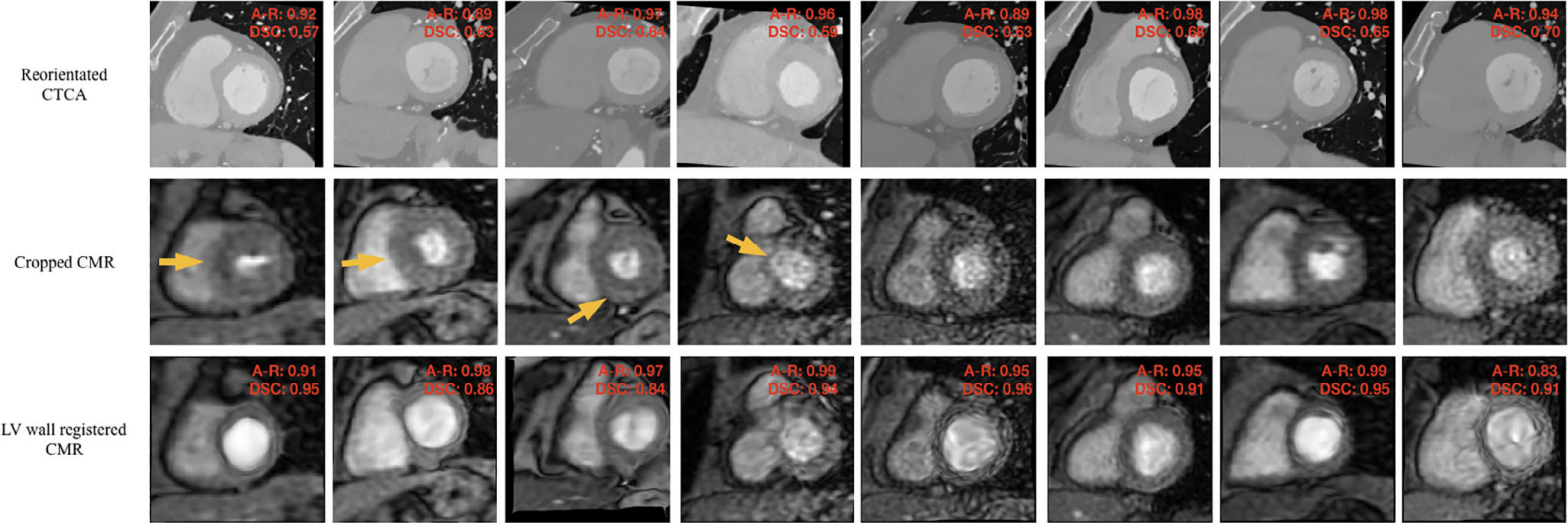
Eight CTCA reorientation results predicted by reorientation STN and corresponding CMR images produced by our pipeline. The first row shows the reoriented CTCA results, and the last two rows show the corresponding CMR images. The abnormal regions are indicated by yellow arrows.

Figure 5 presents the final reorientation results of the CTCA images predicted by the fine‐tuned reorientation STN on the testing cohort. The reorientation STN model produced outcomes where the LV exhibits a near‐circular appearance consistent with the corresponding CMR image, representing reorientation into the target short‐axis view. The DSC between reoriented CTCA and LV wall registered CMR (last row) was numerically higher than that between CTCA and CMR (second row) in each patient.

**FIGURE 6:**
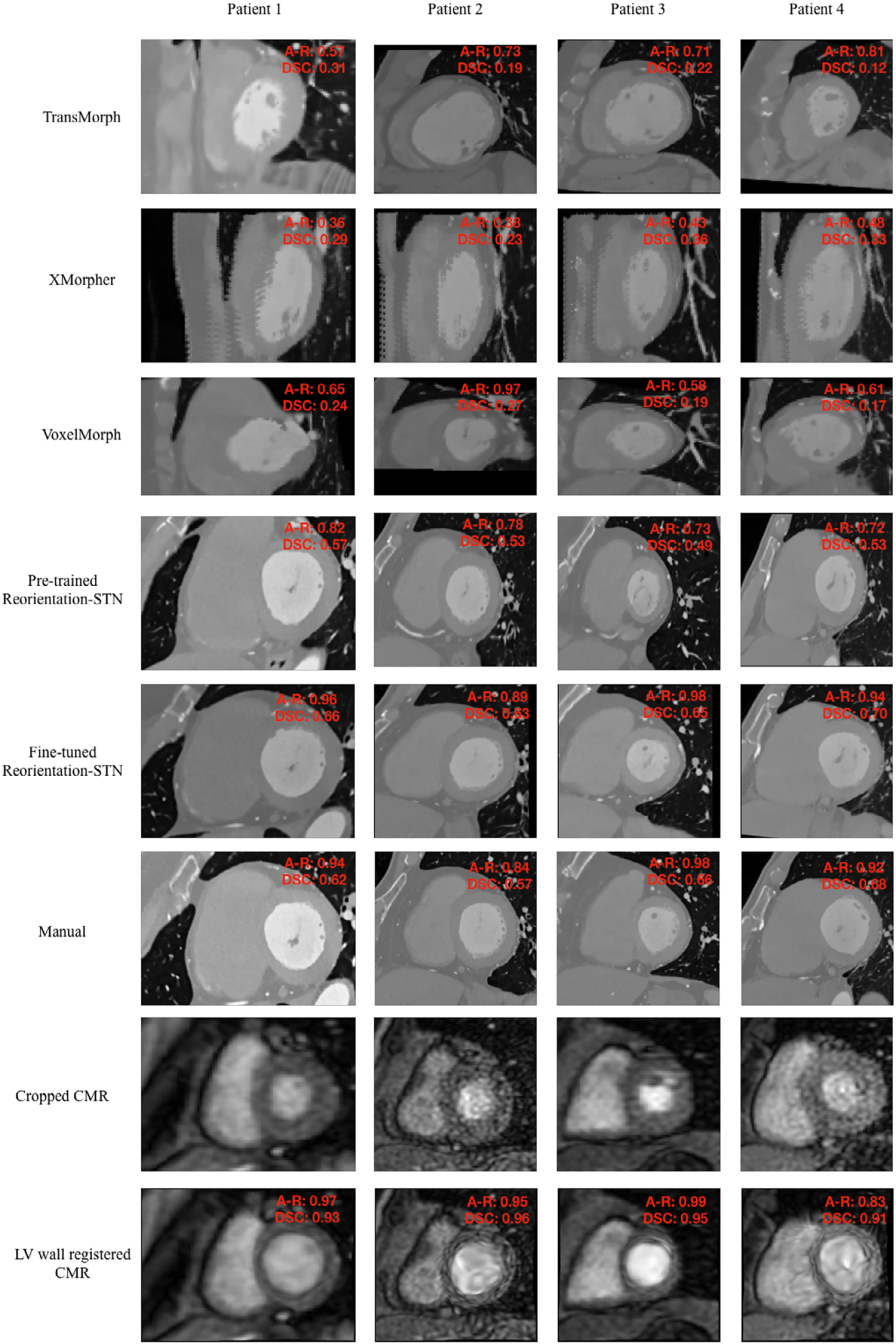
Comparison of results from reorientation STN and the baseline method for four test samples. The first three rows show baseline results, followed by two rows of reorientation STN results and one row of human results. The last two rows display the corresponding CMR images.

Notably, in Figure 5, the first four columns of the second row display representative samples with LV abnormalities, including three cases of LV hypertrophy (Columns 1–3) and one case of LV dilation (Column 4). The abnormal regions are indicated by yellow arrows. Among these, the first and fourth columns demonstrate the highest overlap, while the second and third columns maintain alignment with DSC exceeding 0.8. These findings suggest that our model remains resilient to common LV morphological abnormalities and yields consistent registration performance even in the presence of structural deviations. While the cohort contains only a few cases with hypertrophy or dilation, the representative examples illustrate typical pathological variations. These cases are included in the overall performance analysis, and the results remain consistent with the general cohort findings. Future studies will aim to include additional abnormal cases to allow more comprehensive subgroup evaluations. Figure 6 shows the reorientation results obtained from our framework and those obtained from the benchmark. The reorientation STN model yielded higher AR and DSC than the benchmark in each patient. The fine‐tuned reorientation STN achieved the highest mean AR and DSC among these models and demonstrated performance levels aligned with the manual results produced by human experts, as shown in the fourth row. The LV wall registered CMR attained high DSC with reoriented CTCA in each patient. The results suggested that our proposed framework provides improved performance metrics over the benchmark methods.

**FIGURE 7:**
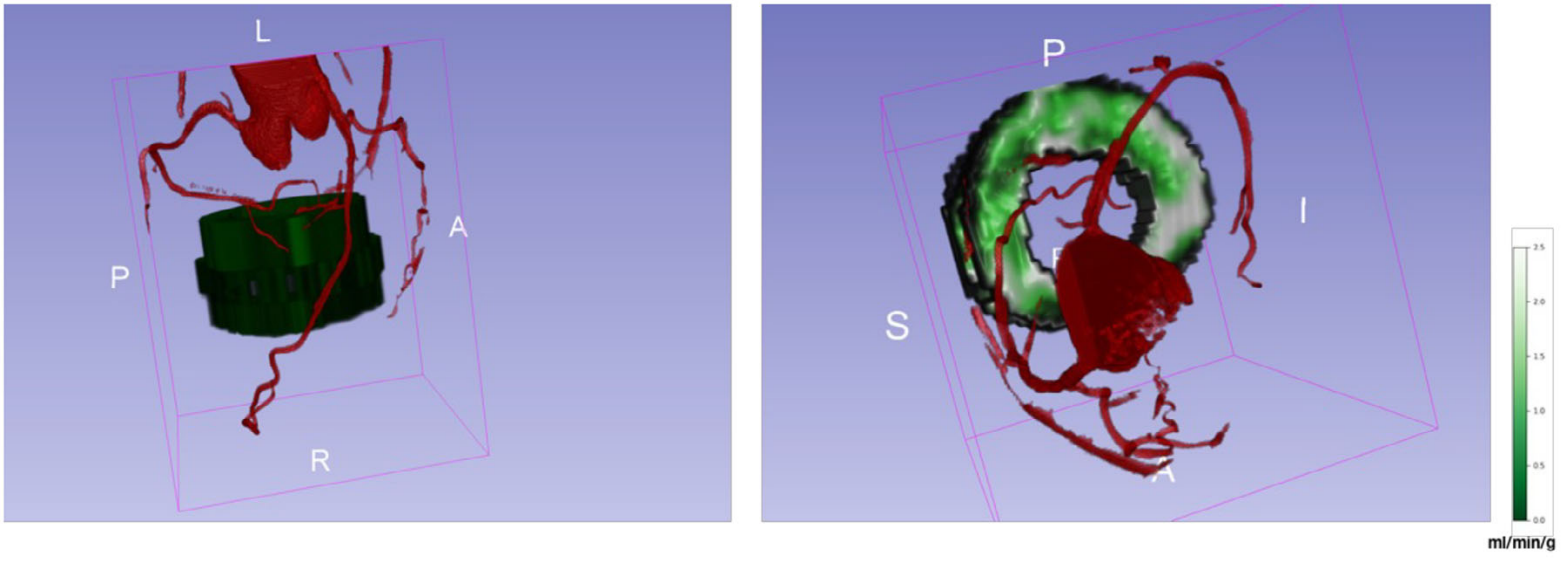
3D image fusion of coronary arteries from reoriented CTCA and perfusion quantification map from LV wall registered CMR on the short‐axis view. The red represents the coronary arteries from reoriented CTCA, and the green represents the perfusion quantification map from LV wall registered CMR.

The last rows in Figures 5 and 6 show the LV wall registered CMR images corresponding to CTCA, which further warps the LV wall thickness on CMR based on that on CTCA images. The resulting LV wall registered CMR images display consistent LV wall thickness across these two modalities. Figure 7 demonstrates the final 3D image fusion for a specific testing case, integrating the coronary arteries from reoriented CTCA with the perfusion quantification map obtained from CMR imaging. Evaluation of coronary artery matching and independent evaluation of the registration between CTCA and CMR detailed results of the ablation study, registration performance across cardiac cycle phases, and the sensitivity analysis of segmentation robustness are provided in the Supplementary Materials.

## 4 DISCUSSION

This study demonstrated a deep learning framework for the 3D fusion of CTCA and perfusion CMR, incorporating automated CTCA reorientation, co‐registration, CMR perfusion quantification, and coronary artery extraction. An interactive 3D rendering of the perfusion quantification map from the CMR images and the coronary arteries from the CTCA images was also produced. Specifically, we proposed a deep learning model called reorientation STN for CTCA reorientation to the short‐axis view. We also combined reorientation STN with image registration and CMR quantification models/methods to achieve the abovementioned framework. Compared with VoxelMorph,^27^ the reorientation STN obtained a numerically higher DSC of 0.66 between reoriented CTCA and CMR and an AR of 0.94 on reoriented CTCA. These findings indicate the performance of our proposed method in reorienting CTCA images while considering anatomical features and cross‐modal correspondence. Our findings contribute to the automated fusion of CTCA and CMR, facilitating AI‐based CTCA and CMR data characterization. Moreover, we used single‐ and cross‐modal losses to train the network for CTCA reorientation. The single‐modal loss served to provide initial orientation guidance, resulting in a final reoriented CTCA that matched the CTCA template. Meanwhile, the cross‐modal loss facilitated the alignment of short‐axis CTCA and corresponding CMR from the same patient. This cross‐modal supervision contributed to the alignment consistency of the CTCA reorientation process. For the cross‐modal loss component, the MSE loss was empirically selected over other cross‐correlation loss candidates, such as MI or NMI. The effectiveness of this choice compared to MI and NMI was evaluated, as shown in the Supplementary Material, Table S2. The improvement of using MSE was statistically insignificant (*p* = 0.051 for NMI and *p* = 0.17 for MI, *p* < 0.05 for significance). Therefore, the MSE, MI, and NMI might be interchangeable for enforcing the cross‐correlation consistency in the current experiment setup. In this study, data augmentation by random rotation was applied only to the CTCA images, as the reorientation STN model was specifically designed to learn CTCA reorientation to the short‐axis view. The corresponding CMR images for each patient were kept fixed and served as supervision during loss calculation. This design was intended to maintain the augmented CTCA maintained consistent alignment with the original CMR reference, thereby reducing potential spatial discrepancies during training.

Moreover, the application of the LV wall nonrigid registration between CMR and CTCA using the nonrigid SDN model accounted for the inherent variations in LV wall thickness observed across different modalities and cardiac phases, yielding a DSC of 0.92 between reoriented CTCA and LV wall registered CMR. Importantly, our pipeline incorporates several components for the inference process: a fine‐tuned reorientation STN model for original CTCA reorientation, a trained nonrigid SDN model for co‐registration between reoriented CTCA and CMR, and a physics‐informed model^23^ for CMR perfusion quantification. To mitigate through‐plane motion in CMR while applying the 2D nonrigid SDN model for CMR and CTCA registration, we aligned the center of the LV circle detected via the Hough transform, given our primary focus on the LV wall structure during registration. Recent advancements^28^, ^29^ in motion correction for CMR images utilizing deep learning methods also offer potential approaches to addressing through‐plane motion in CMR. Furthermore, we employed the Frangi filter technique for coronary artery extraction from CTCA as an illustrative example, considering the labor‐intensive nature of manual annotation. Notably, recent advancements in deep learning, such as the proposed coronary artery segmentation models,^30^, ^31^ offer potential alternatives for clinical application in automating this process. Recent investigations^28^, ^29^, ^32^ have concentrated on motion estimation in perfusion CMR images and motion correction/registration in CMR reconstruction through the development of deep‐learning‐based networks to estimate motion fields rapidly. These studies have reported performance improvements in mitigating motion discrepancies across the temporal dimension. However, these efforts predominantly address motion correction/registration within a single modality (CMR) and less frequently address multimodal registration in cardiac imaging, which is important for comprehensive CAD evaluation. Coronary artery stenosis and myocardial ischemia are critical in evaluating CAD, typically assessed using CTCA and CMR perfusion imaging. Integrating morphological and functional imaging modalities has been recognized as a valuable approach for conditions amenable to revascularization.^1^, ^33^, ^34^ Prior research has demonstrated that 3D fusion imaging can facilitate the anatomical correlation between stress‐induced myocardial perfusion deficits and their corresponding causative lesions.^3^, ^4^, ^35^, ^36^ This study presents a fully automatic deep‐learning‐based framework. Automating the fusion of coronary arteries in cardiac CTCA and LV wall perfusion quantification in CMR imaging has the potential to reduce clinical workload. Furthermore, recent advancements in medical and cardiac anatomical segmentation^37^, ^38^, ^39^ have introduced efficient alternative tools for acquiring masks for various cardiac structures, such as the LV, LV wall, RV, and RV wall. Utilizing these segmentation tools, RV wall registration between CMR and CTCA can be implemented using the nonrigid SDN model.

Our research presents a fully automated, deep‐learning‐based cross‐modal image fusion framework for cardiac CTCA and CMR, demonstrating increased time efficiency and processing speed compared to conventional methods while preserving crucial anatomical details. This framework facilitates spatial correlation between coronary artery anatomy and myocardial tissue properties, relevant for thorough cardiovascular evaluation. While our study did not focus on specific diseases, the observed alignment may support clinical applications like improved treatment planning and better identification of ischemic areas.^4^, ^40^ These findings align with the evolving role of multimodal fusion and AI in cardiovascular imaging and patient care.^4^

In this study, the registration performance measured by the Hausdorff distance (HD) was generally maintained below 10 mm. Although no universally accepted threshold defines clinically significant misalignment in cardiac imaging, prior research suggests that spatial discrepancies around 10–20 mm may affect quantitative assessments and diagnostic decisions.^41^ Thus, maintaining HD within 10 mm in our work suggests a potential for preserving anatomical alignment critical for clinical evaluation, particularly in regions like the LV and myocardium. Nevertheless, the clinical significance of such performance may vary with specific applications, and further studies involving larger cohorts are necessary to better establish precise thresholds and their practical impact. Besides, we focused on the end‐diastolic CMR frame, which exhibits minimal cardiac motion and is commonly used for cardiac imaging. Future work could consider multiphase analysis or motion‐correction techniques to further evaluate robustness, especially for high or irregular heart rates. Our coronary branch‐level analysis (Supplementary Results) showed numerically smaller misregistration for LAD (3.1 ± 0.3 mm) than RCA (5.9 ± 1.4 mm), consistent with reports that RCA is more difficult to align due to motion and projection effects.^42^ The overall error (4.5 ± 0.8 mm) is comparable to cross‐modality CTCA–CMR surface registration (∼4.1 mm),^43^ reflecting the challenges inherent in cross‐modality and 3D‐to‐2D evaluation. As few studies report per‐vessel errors in millimeters; our LAD/RCA statistics provide data regarding vessel‐specific trends consistent with prior observations.

There were a few limitations of the proposed method. A primary limitation arose from using a 3‐slice CMR model, which did not encompass the entire heart as does the whole heart imaging approach of CTCA. This method may lead to potential misalignments across different slices due to its limited coverage. Future work could improve upon this by incorporating 3D static CMR data as a reference during the registration of CMR and CTCA images. Another major limitation of our study was that CTCA data were acquired at discrete cardiac phases and not throughout the cardiac cycle. In contrast, CMR perfusion data were from a dynamic sequence acquired at different points in the cardiac cycle. As a result, precise temporal synchronization between the two modalities was not fully established. Therefore, our registration and fusion analysis used the CMR frame that most closely matched the CTCA acquisition phase. However, residual misalignment due to cardiac motion may persist, particularly in regions with rapid motion, such as the right coronary artery. This temporal mismatch may introduce spatial discrepancies in the fused images, especially in patients with high heart rates or arrhythmias. Future studies using multiphase CTCA or advanced motion correction techniques might help enhance alignment precision. Additionally, our registration model primarily targeted the LV and myocardial wall regions without including other anatomical structures, such as the RV. The dataset consisted of a limited number of abnormal cases and did not cover the full range of potential cardiac morphological variations. While our results indicated consistent performance metrics in common LV abnormalities like hypertrophy and dilation, the model's performance was influenced by the relatively high contrast of the LV region. This dependence may restrict its robustness in areas with lower contrast or more complex anatomy. Future studies should aim to validate and enhance the model's generalizability across a broader spectrum of cardiac pathologies, including more extensive morphological abnormalities and other cardiac chambers. During the evaluation phase, we acknowledge that the perturbation strategy employed in the segmentation‐noise study may not fully capture realistic segmentation errors. Future work could explore more representative perturbation models, such as boundary erosions/dilations or mislabeled structures, to better assess robustness under practical conditions. The limited sample size and lack of external validation across imaging centers and scanner models may restrict the generalizability of our findings. Expanding the dataset and conducting cross‐center validation would further evaluate its performance across different patient cohorts and protocols, which should be performed in future studies. Moreover, exploring the applicability of the framework in other cardiac imaging modalities, such as echocardiography or nuclear imaging, would extend its potential adaptation in clinical practice. Future work will include a prospective user study to assess workflow integration, including sample size, task protocols, and timing metrics.

## 5 CONCLUSION

Our research developed a fully automated fusion framework for cardiac CTCA and CMR imaging, which achieved precise alignment of the LV wall and coronary artery structures on CMR and CTCA images in the short‐axis view.

## CONFLICT OF INTEREST STATEMENT

The authors declare no conflicts of interest.

## Supporting information



Supporting information

## Data Availability

Data sharing is not applicable due to confidential patient data protected under ethical restrictions. For inquiries, contact the corresponding author.
