# Supplementary material for: A deep‐learning‐based pipeline for automatic fusion of CT coronary angiogram and stress perfusion CMR
Source: Med Phys. 2026 Apr 10;53(4):e70420. doi: 10.1002/mp.70420 (PMC13067337; doi:10.1002/mp.70420)
Supplement: Supplementary file 1 — Supporting information [file MP-53-0-s001.docx]

**Methods:** **Performance and statistics**

Quantitative evaluation of CTCA reorientation and registration to CMR

To investigate the impact of different loss functions on registration performance, we conducted an ablation study comparing mean squared error (MSE), mutual information (MI), and normalized mutual information (NMI) losses within our ResNet-STN framework. The quantitative results are summarized in Tables S-1 and S-2. In Table S-1, our method showed significantly improved performance compared with XMorpher, TransMorph, and VoxelMorph. Meanwhile, in Table S-2, our findings indicated that the model trained with MSE loss yielded the highest mean values for both metrics (AR: 0.94; DSC: 0.66). However, its performance was not statistically different from that of MI (p=0.33 for AR, p=0.17 for DSC) and NMI (p=0.27 for A-R, p=0.05 for DSC) based on the Wilcoxon signed-rank test.

Evaluation of coronary artery matching between CTCA and CMR

To assess the misalignment in coronary artery matching between CTCA and CMR, we calculated the Euclidean distance between the coronary arteries in the reoriented CTCA and the corresponding CMR images (Figure 4 c). For branch-level analysis, we measured registration errors in the three major coronary territories: the left anterior descending (LAD), left circumflex (LCX), and right coronary (RCA) arteries. In the test cohort, the overall mean distance was 4.5 ± 0.8 mm. At the zonal level, LAD demonstrated smaller errors (3.1 ± 0.3 mm) compared with RCA (5.9 ± 1.4 mm). Reliable measurements could not be obtained for LCX due to limited visibility on the corresponding CMR slices, and thus LCX values are not reported.

Independent evaluation of the registration between CTCA and CMR

To further validate our model against the benchmark, we performed a qualitative assessment with a panel comprising one senior cardiac radiologist with over ten years of experience in cardiac imaging, one clinical radiologist, and two MRI scientists with four years of experience each. To further validate our model in comparison to the benchmark, we conducted a qualitative assessment using a panel of one senior cardiac radiologist with over ten years of experience in cardiac imaging, one clinical radiologist and two MRI scientists of four-year experience. Each human expert independently examined the 3 kinds of reoriented CTCA images for each patient on the testing cohort from 3 different methods, including VoxelMorph, pre-trained Reorientation-STN and fine-tuned Reorientation-STN.

The results of the qualitative assessment revealed that the proposed model Reorientation-STN achieved the highest average scores of 3.27 and 3.58 among the evaluated methods (mean score: 1.65, 3.27, 3.58). This performance reflects accurate reorientation and alignment of main anatomical structures, minimal artifacts, and realistic deformations, making it highly suitable for diagnostic and treatment planning purposes. The inter-rater reliability of the fine-tuned Reorientation-STN, measured using the intraclass correlation coefficient (ICC), was 0.89, demonstrating strong agreement among the reviewers.

Registration Performance Across Cardiac Cycle Phases

We evaluated registration accuracy at different cardiac phases, including end-diastole (ED), mid-diastole, and end-systole (ES), using DSC as the primary metric. As summarized in Table S-3, the proposed method achieved slightly higher DSC at the ED phase (global DSC = 0.66) and a higher expert interpretability score (3.58) compared with ES, indicating more accurate alignment during diastole.

Sensitivity Analysis of Segmentation Robustness

To assess the impact of segmentation inaccuracies on registration and fusion quality, we systematically introduced varying levels of random noise into the segmentation masks and evaluated the resulting performance. As summarized in Table S-4, increasing the noise level resulted in a gradual decrease in both Dice Similarity Coefficient (DSC) and an increase in Hausdorff Distance (HD). Specifically, even at higher noise levels, the registration and fusion results remained within an acceptable range, indicating that our method is reasonably robust to segmentation errors. These findings demonstrate that the overall performance of the framework is not overly sensitive to moderate segmentation inaccuracies, supporting its practical applicability in clinical scenarios.

In addition to noise analysis, we provided a quantitative measure of robustness against realistic segmentation errors by applying morphological erosion and dilation (with 1 and 2 pixels) to the segmentation masks. This analysis demonstrated stability across the perturbations, though with a noticeable reduction in accuracy. Specifically, the maximum observed drop in DSC was 0.11 (under 2-pixel erosion), while the maximum increase in HD was 2.1 mm (under 2-pixel erosion), suggesting acceptable performance margins against realistic boundary inaccuracies (Table S-5).

All scripts required for inference, along with the trained model weights, will be publicly released upon publication to facilitate reproducibility of our results.

**Tables**

**Table S-1.** Statistical results of quantitative evaluation for proposed method and benchmark models, including mean ± SD, 95% confidence intervals, pairwise differences (Proposed − Baseline), and p-values (Wilcoxon signed-rank test). AR = aspect ratio; DSC = Dice Similarity Coefficient.

| Metric | Method / Comparison |  |
| --- | --- | --- |
| AR | Summary of methods | Value, mean ± SD (95% CI) |
|  | XMorpher | 0.42 ± 0.06 (0.35 - 0.48) |
|  | TransMorph | 0.68 ± 0.09 (0.63 - 0.74) |
|  | VoxelMorph | 0.73 ± 0.13 (0.65 - 0.81) |
|  | ResNet-STN (ours) | 0.94 ± 0.03 (0.91 - 0.95) |
|  | Pairwise comparisons | Difference reflects improvement |
|  | ResNet-STN vs XMorpher | 0.52 ± 0.04 (0.47 - 0.55), p=0.001 |
|  | ResNet-STN vs TransMorph | 0.26 ± 0.08 (0.19 - 0.31), p=0.001 |
|  | ResNet-STN vs VoxelMorph | 0.21 ± 0.12 (0.12 - 0.28), p=0.001 |
| DSC | Summary of methods | Value, mean ± SD (95% CI) |
|  | XMorpher | 0.27 ± 0.06 (0.22 - 0.31) |
|  | TransMorph | 0.21 ± 0.05 (0.18 - 0.24) |
|  | VoxelMorph | 0.23 ± 0.04 (0.19 - 0.26) |
|  | ResNet-STN (ours) | 0.66 ± 0.04 (0.64 - 0.68) |
|  | Pairwise comparisons | Difference reflects improvement |
|  | ResNet-STN vs XMorpher | 0.39 ± 0.06 (0.33 - 0.41), p=0.001 |
|  | ResNet-STN vs TransMorph | 0.45 ± 0.05 (0.42 - 0.48), p=0.001 |
|  | ResNet-STN vs VoxelMorph | 0.43 ± 0.05 (0.40 - 0.46), p=0.001 |

**Table S-2.** Ablation results of our ResNet-STN with Mean Squared Error (MSE), Mutual Information (MI), and Normalized Mutual Information (NMI) loss functions, including mean ± SD, 95% confidence intervals, pairwise differences, and p-values (Wilcoxon signed-rank test).

| Metric | Method / Comparison |  |
| --- | --- | --- |
| AR | Summary of methods | Value, mean ± SD (95% CI) |
|  | MSE | 0.94 ± 0.09 (0.89 - 0.99) |
|  | MI | 0.89 ± 0.15 (0.83 - 0.97) |
|  | NMI | 0.88 ± 0.12 (0.82 - 0.95) |
|  | Pairwise comparisons | Difference reflects improvement |
|  | MSE vs MI | 0.05 ± 0.14 (-0.04 - 0.14), p=0.33 |
|  | MSE vs NMI | 0.06 ± 0.12 (-0.01 - 0.14), p=0.27 |
| DSC | Summary of methods | Value, mean ± SD (95% CI) |
|  | MSE | 0.66 ± 0.07 (0.63 - 0.68) |
|  | MI | 0.61 ± 0.12 (0.55 - 0.68) |
|  | NMI | 0.59 ± 0.09 (0.54 - 0.64) |
|  | Pairwise comparisons | Difference reflects improvement |
|  | MSE vs MI | 0.05 ± 0.12 (-0.03 - 0.12), p=0.17 |
|  | MSE vs NMI | 0.07 ± 0.10 (0.012 - 0.14), p=0.05 |

**Table S-3.** Registration Accuracy and Expert Evaluation Across Cardiac Cycle Phases. Abbreviations: DSC = Dice Similarity Coefficient; ED/ES = End-diastole/End-systole.

| CMR Frame (Cardiac Phase) | Global DSC | *Expert Score (1-4)* |
| --- | --- | --- |
| ED (End-diastole) | 0.66 | 3.58 |
| ES (End-systole) | 0.61 | 2.83 |
| *Mid-diastole* | 0.64 | 3.21 |

**Table S-4.** Sensitivity Analysis of Segmentation Robustness: Impact of increasing random noise in the segmentation mask on registration and fusion quality. Noise level indicates the percentage of voxels randomly flipped in the mask. DSC = Dice Similarity Coefficient; HD = Hausdorff Distance (mm).

| Noise Level (%) | DSC | HD (mm) |
| --- | --- | --- |
| 0 (Original) | 0.66 | 6.3 |
| 1 | 0.63 | 6.7 |
| 3 | 0.61 | 7.0 |
| 5 | 0.59 | 7.5 |
| 10 | 0.54 | 8.2 |

**Table S-5.** Sensitivity Analysis of Segmentation Robustness: Impact of Morphological Perturbations on registration and fusion quality. Perturbation Level indicates the kernel radius applied in pixels. DSC = Dice Similarity Coefficient; HD = Hausdorff Distance (mm).

| Perturbation Type | *Level (pixels)* | DSC | HD (mm) |
| --- | --- | --- | --- |
| Original | 0 | 0.66 | 6.3 |
| Erosion (Under-segmentation) | 1 | 0.59 | 7.7 |
|  | 2 | 0.55 | 8.4 |
| Dilation (Over-segmentation) | 1 | 0.62 | 7.3 |
|  | 2 | 0.57 | 8.1 |
